# Kinetics and protective role of autophagy in a mouse cecal ligation and puncture-induced sepsis

**DOI:** 10.1186/cc12839

**Published:** 2013-07-24

**Authors:** Waka Takahashi, Eizo Watanabe, Lisa Fujimura, Haruko Watanabe-Takano, Hiroyuki Yoshidome, Paul E Swanson, Takeshi Tokuhisa, Shigeto Oda, Masahiko Hatano

**Affiliations:** 1 Department of Emergency and Critical Care Medicine, Graduate School of Medicine, Chiba University, 1-8-1 Inohana, Chuo-ku, Chiba City 260-8670, Japan; 2 Biomedical Research Center, Chiba University, 1-8-1 Inohana, Chuo-ku, Chiba City 260-8670, Japan; 3 Department of Biomedical Science, Graduate School of Medicine, Chiba University, 1-8-1 Inohana, Chuo-ku, Chiba City 260-8670, Japan; 4 Department of General Surgery, Graduate School of Medicine, Chiba University, 1-8-1 Inohana, Chuo-ku, Chiba City 260-8670, Japan; 5 Department of Pathology, University of Washington, School of Medicine, Seattle, WA, USA; 6 Department of Developmental Genetics, Graduate School of Medicine, Chiba University, 1-8-1 Inohana, Chuo-ku, Chiba City 260-8670, Japan

## Abstract

**Introduction:**

It is not well understood whether the process of autophagy is accelerated or blocked in sepsis, and whether it is beneficial or harmful to the immune defense mechanism over a time course during sepsis. Our aim was to determine both the kinetics and the role of autophagy in sepsis.

**Methods:**

We examined autophagosome and autolysosome formation in a cecal ligation and puncture (CLP) mouse model of sepsis (in C57BL/6N mice and GFP-LC3 transgenic mice), using western blotting, immunofluorescence, and electron microscopy. We also investigated the effect of chloroquine inhibition of autophagy on these processes.

**Results:**

Autophagy, as demonstrated by increased LC3-II/LC3-I ratios, is induced in the liver, heart, and spleen over 24 h after CLP. In the liver, autophagosome formation peaks at 6 h and declines by 24 h. Immunofluorescent localization of GFP-LC3 dots (alone and with lysosome-associated membrane protein type 1 (LAMP1)), as well as electron microscopic examination, demonstrate that both autophagosomes and autolysosomes are increased after CLP, suggesting that intact autophagy mechanisms operate in the liver in this model. Furthermore, inhibition of autophagy process by chloroquine administration immediately after CLP resulted in elevated serum transaminase levels and a significant increase in mortality.

**Conclusions:**

All autophagy-related processes are properly activated in the liver in a mouse model of sepsis; autophagy appears to play a protective role in septic animals.

## Introduction

Sepsis is a life-threatening condition that causes multiple organ failure and shock. It initiates host immune, inflammatory, and coagulation responses that cause tissue injury, hypoxia and organ dysfunction and predispose patients to refractory infection [1]. Despite advances in critical care treatment and increased understanding of the pathophysiology of sepsis, the mortality rate of affected patients remains high (40 to 60%) even in developed countries [2]. This is particularly important as the incidence of sepsis increases in an expanding aged population with treatment-resistant infections and compromised immune function. Excessive levels of pro-inflammatory cytokines and chemokines cause subsequent accumulation of neutrophils and immune cells, which release reactive oxygen species and proteases. These mediators and dysoxia induce cell death and subsequent organ dysfunction [3].

Autophagy is a bulk intracellular degradation system responsible for disposal of damaged and senescent organelles and denatured proteins using lysosomal processes [4-6]. Autophagy involves the formation of specialized double-membrane vesicles - autophagosomes - which envelop target cytosolic materials and then secondarily fuse with lysosomes, followed by enzymatic degradation of both the inner membrane of the autophagosome and its contents. The resultant structure is a single-membrane organelle, the autolysosome. The electron microscopic appearance of autolysosomes as contents further degrade over time forms the morphologic spectrum of heterolysosomes. Macromolecules resulting from this process are recycled to the cytoplasm and are used for anabolic pathways and energy production [7,8].

Under physiological conditions, autophagy plays important roles in pre-implantation embryonic development, survival during neonatal starvation, and cell differentiation of lymphocytes, erythrocytes, and adipocytes [9-13]. Autophagy is also crucial to the maintenance of terminally differentiated cells, such as neurons [14-16]. Autophagy is induced beyond basal levels in response to environmental signals (such as fasting), hormones (such as glucagon), and microbial pathogens [17-19] and aids cell survival by producing energy during starvation, and eliminating pathogens from infected cells [6,8,19].

Recent studies have demonstrated that autophagy is also induced in patients with sepsis and in the clinically relevant cecal ligation and puncture (CLP) animal model of sepsis [20-27]. Autophagic structures can be identified by electron microscopy in livers of patients who died of sepsis, and the number of these structures is significantly greater than that seen in non-septic control patients [28]. Autophagy is also induced in the heart and lungs in the CLP model [20,24]. However, it is not yet well-defined as to what extent the process of autophagy is completed, whether it is accelerated, or indeed, whether it is at times partially or completely blocked prior to fusion of autophagosomes with lysosomes. It is also not known with clarity whether autophagy is generally beneficial or harmful to the immune defense mechanism or other cell functions in sepsis.

In this study, we investigated both the kinetics of autophagy and importance of this process to survival in sepsis using a mouse CLP model. We found that the entire autophagy system (from autophagosome formation to degradation of lysosomal contents) functions in the CLP mouse liver over a 24-h post-CLP observation period, and demonstrated that inhibition of autophagy results in hepatocyte damage and decreased survival compared to sham-treated control animals. We use these observations to discuss the role of autophagy in sepsis.

## Materials and methods

### Animals

Male C57BL/6N (6- to 8-week-old) mice and green fluorescent protein (GFP)- microtubule-associated protein light chain 3 (LC3) transgenic mice (C57BL/6 background; 6- to 8-week-old) were acclimated to a 12-h day/night cycle under specific pathogen-free conditions with food at least 1 week before experiments. All experimental procedures were approved by the Institutional Animal Care and Use Committees of Chiba University and were in compliance with the National Institute of Health guidelines.

### Cecal Ligation and Puncture (CLP) model

Sepsis was induced by CLP as described previously [29,30]. Briefly, mice were anesthetized with isoflurane and after laparotomy, the cecum was ligated with a 3-0 silk tie and punctured with a 25-gauge needle at two sites, followed by expression of a small amount of fecal material into the peritoneal cavity. After surgery, 2 ml of 0.9% saline was injected subcutaneously. Sham-operated mice were treated with the same procedure, but without cecum ligation and puncture. No antibiotics or analgesics were used, and mice were food-deprived but had free access to water postoperatively. In selected animals, chloroquine (60 mg/kg) was injected intra-peritoneally 1 h after the operation [31]. Mice were sacrificed at indicated time points after treatment and tissue samples were taken for analysis. Survival was examined after chloroquine administration (n = 18). Mice were observed every day by animal caretakers who were blinded to the treatment arms, and the mice were sacrificed when they were moribund.

### Western blot analysis

Total proteins were prepared from mouse organs. Each tissue was lysed in 2 × SDS sampling buffer (1.245 M Tris-HCl at pH 6.8 containing 10% glycerol, 4.6% sodium dodecyl sulfate, 10% 2-mercaptoethanol, and 0.04% bromophenol blue). Extracts were homogenized on ice and boiled for 5 minutes; these were then centrifuged at 10,000 × *g* for 10 minutes at room temperature, and the supernatants were obtained as total protein.

Equal amounts of protein were separated by SDS-polyacrylamide gel electrophoresis and transferred to polyvinylidene difluoride membrane. The membranes were subsequently incubated with 5% nonfat dry milk in Tris-buffered saline (TBS) containing 0.1% Tween-20 (TBS-T) for 1 h at room temperature. Antibodies were added and incubated overnight at 4°C in TBS-T. The following primary antibodies were used: rabbit polyclonal anti-LC3B (Sigma-Aldrich, St. Louis, MO; 1:1000), mouse monoclonal anti-β tubulin (clone E7; Developmental Studies Hybridoma Bank, Iowa city, IA, 1:1000), rabbit polyclonal anti-p62 (DakoCytomation, Glostrup, Denmark, 1:500). Membranes were washed 3 times in TBS-T and subsequently incubated with peroxidase conjugated secondary antibodies (goat anti-rabbit IgG: Jackson Immuno Research, West Grove, PA, 1:2000; goat anti-mouse IgG: Jackson Immuno Research, 1:2000). Blots were washed three times with TBS-T and once with TBS, and the signal was then detected using enhanced chemiluminescence (ECL-Plus) reagent (GE Healthcare, Piscataway, NJ, USA). Band images were scanned and densitometric analysis was performed using NIH Image software (Bethesda, MD, USA). Quantification data, evaluated by band-intensity of LC3-I and -II, were normalized to that of β-tubulin. Results are representative of seven independent experiments.

### Real-time quantitative reverse transcription Polymerase Chain Reaction (PCR)

Total RNA was extracted from the liver tissue using RNeasy Mini Kit (Qiagen Inc., Valencia, CA, USA), and single-stranded cDNA was synthesized with SuperScript VILO cDNA Synthesis Kit (Life Technologies, Grand Island, NY). The expression of LC3 mRNA was determined by quantitative real-time PCR with the cDNA, using a SYBR Green PCR Master Mix (Life Technologies) and run on the StepOne Real-Time PCR System (Life Technologies). The mRNA levels were measured as the relative ratio to the β-actin mRNA levels. The quantification data were analyzed with the LightCycler analysis software as described [32]. The following primers were used: *LC3* sense: (5'-CGATACAAGGGGGAGAAGACA-3'), *LC3* antisense: (5'-ACTTCGGAGATGGGAGTGGA-3'), *β-actin* sense: (5'-CCAGCCTTCCTTCTTGGGTAT-3'), *β-actin* antisense: (5'-TGGCATAGAGGTCTTTACGGATGT-3').

### Immunofluorescent microscopy

Mice were transcardially perfused with 4% paraformaldehyde (PFA) in phosphate buffer. Tissues of interest were removed and were further fixed with 4% PFA at 4°C overnight. Samples were then placed in 15% sucrose in PBS at 4°C for 4 h; this was then exchanged for 30% sucrose in PBS, and incubation continued at 4°C overnight. The tissues were frozen in optimum cutting temperature (OCT) compound and sectioned serially into 4-μm-thick sections using a cryostat [33]. Samples were kept frozen at -80°C until used.

For immunofluorescence, sections were stained using rabbit polyclonal anti-lysosome-associated membrane protein type 1 (LAMP1) (Abcam, Cambridge, UK; 1:1000). Cy3-conjugated goat anti-rabbit immunoglobulin G (IgG) (H+L) was used as a secondary antibody (Jackson Immuno Research; 1:1000 dilution). All fluorescence images were digitally acquired with an Olympus Fluoview 1000 confocal microscope (Olympus America, Inc., Center Valley, PA, USA).

### Electron microscopic analysis

Samples were fixed with 2% PFA, and 2% glutaraldehyde in 0.1 M phosphate buffer, pH 7.4, at 4°C overnight. After fixation and dehydration, 70-nm sections were prepared with a diamond blade, using an ultramicrotome (ULTRACUT UCT; Leica) and mounted on metal grids. These were stained with 2% uranyl acetate and secondarily stained with lead solution and examined with a transmission electron microscope (JEM-1200EX; JEOL Ltd., Tokyo, Japan). Specimens were examined as previously described [28]. Briefly, a minimum of 8 to 10 random fields (to minimize unintended sampling bias) were examined at 2,500× magnification for evidence of autophagy or cell injury/death, and the number of autophagosomes and autolysosomes in each 2,500× image was counted. The mean ± SD per 50 images from each mouse was calculated and the data from different groups were compaired (CLP (n = 3) versus sham (n = 3)).

In the present study, autophagosomes were defined as double-membrane structures that enclosed cytoplasm with damaged organelles in various stages of degradation; double membrane structures enclosing only materials that resembled background cytoplasm were not counted. Autolysosomes were defined as single membrane vesicles with cytoplasmic or organellar debris in various stages of degradation (usually less obviously the remains of specific organelles compared with autophagosomeal contents). Lysosomes with amorphous electron-dense material (heterolysosomes) were not counted. Because initial counting of images was performed by the same investigator who created the images (EW), the possibility of unintended bias was mitigated by providing the same set of images in a blinded fashion to a second investigator (PES). When results of initial counting differed markedly between observers, relevant images were re-evaluated and discrepancies were resolved. The 2,500× survey images used in this analysis represent approximately 3,000 square microns of tissue, each containing 5 to 8 hepatocytes and a variable complement of Kupffer cells, stellate cells, sinusoidal endothelial cells and inflammatory cells. Only the hepatocytes were counted.

### Histological analysis

Liver tissue specimens were obtained and sections of formalin-fixed paraffin-embedded liver samples were stained with hematoxylin-eosin to assess the degree of liver injury.

### Analysis of transaminase to assess liver injury

Blood samples were obtained from the tail arteries of mice (n = 7). Serum aspartate aminotransferase (AST) and alanine aminotransferase (ALT) activity was quantified using the transaminase C-ll test (Wako Chemical, Tokyo, Japan).

### Statistical analysis

All data were analyzed for statistical significance using the Mann-Whitney test or one-way analysis of variance, and individual group means were then compared with a Student-Newman-Keuls test. All data were expressed as the mean ± SD using the statistical software program PRISM (GraphPad Software, San Diego, CA, USA). Overall survival was calculated using the Kaplan-Meier method, and comparisons were evaluated using the log-rank test. Data were analyzed using SPSS 21.0 software. *P*-values <0.05 were considered to be statistically significant.

## Results

### Autophagosome formation in various organs after cecal ligation and puncture in mice

**Figure 1:**
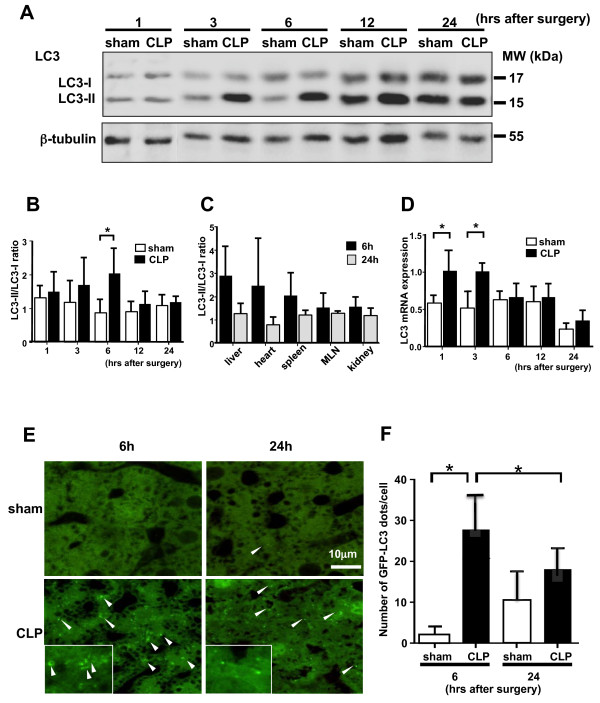
**Cecal ligation and puncture (CLP) induces autophagy in the liver of CLP model mice.** For **B-D** and **F**, data are expressed as mean ± SD; data were analyzed for statistical significance using the Mann-Whitney test. **(A)** Western blotting analysis of microtubule-associated protein light chain 3 (LC3) in the liver. Sepsis was induced by CLP. Liver samples were prepared from sham-operated and CLP mice at each indicated time point post surgery. Results are representative of seven independent experiments. β-tubulin served as standard. **(B)** The ratio between the levels of LC3-II and LC3-I at each time point. ^*^P <0.05, as compared to sham-operated group (n = 7/group). **(C)** The ratio of LC3-II/LC3-I expression in various organs at 6 and 24 h after CLP. Values in CLP mice are expressed as fold-change relative to each sham-operated group. MLN, mesenteric lymph node. **(D)***LC3* mRNA expression in the liver at each time point assessed by real-time PCR. ^*^P <0.05, as compared to sham-operated group (n = 7/group). **(E)** Confocal images of liver samples obtained from GFP-LC3 transgenic mice. Green-fluorescing GFP-LC3 dots were present in the cytosol indicated by the arrow head. **(F)** The number of GFP-LC3 dots per cellular confocal image was quantified. Differences in the number of GFP-LC3 dots in CLP mice at 6 h compared with sham-operated mice at 6 h and CLP mice at 24 h were statistically significant (^*^P <0.05; n = 50 cells /animal; n = 4 animals).

Autophagy is induced under various types of stress. Autophagosome formation, the initial step of autophagy, can be assessed by following the conversion of cytosolic LC3-I protein (17 kDa) to autophagosomal membrane-bound LC3-II (15 kDa) by SDS-PAGE. We examined induction of autophagy after CLP in various organs. Liver, spleen, heart, mesenteric lymph nodes, and kidney were isolated at 1, 3, 6, 12, or 24 h after CLP and autophagosome formation in these organs evaluated by western blotting. In the sham-operated mice, the LC3-II/LC3-I ratio slightly increased over the time course following surgery and declined by 24 h after surgery (Figure 1A). In the liver, a significant increase in LC3-II/LC3-I ratio was observed at 6 h after CLP, and the ratio returned to basal levels by 24 h (Figure 1A,B). The same tendency was observed in the heart and spleen (Figure 1C). In contrast, minimal or no change was seen in the ratio in the mesenteric lymph node and kidney over the time course following CLP (Figure 1C).

Since the liver is one of the critical organs in sepsis and induction of autophagy was greatest in the liver in this study, based on LC3-II/LC3-I ratios, we focused on the liver in subsequent analyses. To further investigate LC3 induction, we examined *LC3* mRNA expression in the liver. The expression significantly increased 1 and 3 h after CLP compared to the sham group (Figure 1D), indicating that the total amount of LC3 protein in the liver increased at a transcriptional level and was then converted to LC3-II post-transcriptionally. Autophagosome formation was also examined using GFP-LC3 transgenic mice. In these mice, autophagosomes are visualized as cytoplasmic GFP-LC3 dots by confocal microscopy. In agreement with the western blotting data, CLP induced an increase in GFP-LC3 dots; the number peaked at 6 h (27.6 ± 9.1/cell) and then decreased by 24 h (18.3 ± 5.8/cell) in the liver (Figure 1E,F). No significant increase in GFP-LC3 dots was observed in the sham-operated group.

### Completion of autophagy induction in the liver after CLP

**Figure 2:**
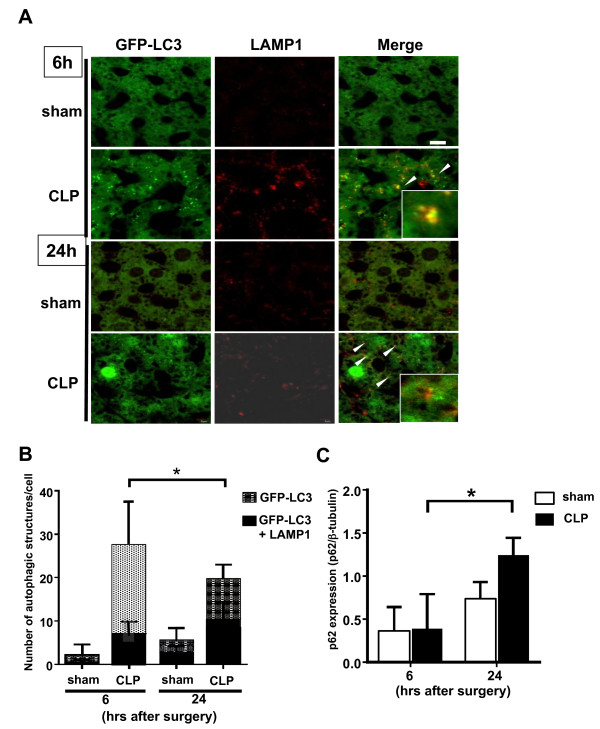
**Co-localization of green fluorescent protein (GFP)-Microtubule-associated protein light chain 3 (LC3) dots with lysosome-associated membrane protein type 1 (LAMP1) in the liver after cecal ligation and puncture (CLP). (A)** Confocal images of liver samples obtained from GFP-LC3 transgenic mice. Green-fluorescing GFP-LC3 dots were present in the cytosol. LAMP1 was stained using Cy3-conjugated IgG secondary antibodies. Merged images demonstrate co-localization of GFP-LC3 dots and LAMP1. **(B)** The number of GFP-LC3 or GFP-LC3 + LAMP1 dots per cellular confocal image was quantified at 6 and 24 h after CLP. All data are expressed as mean ± SD. Data were analyzed for statistical significance using the Mann-Whitney test. Differences in the number of GFP-LC3 or GFP-LC3 dots in CLP mice at 6 h compared with CLP mice at 24 h were statistically significant (^*^P <0.05; n = 50 cells /animal; n = 4 animals). **(C)** Relative expression of p62 protein in the liver at 6 and 24 h after sham or CLP operation. The amount of p62 protein was normalized to that of β-tubulin by evaluation of band intensity from western blotting. All data were expressed as the mean ± SD. Data were analyzed for statistical significance using the Mann-Whitney test (^*^P <0.05; n = 5 in each group).

An increase in autophagosome numbers does not necessarily infer completion of the autophagy process. The autophagosome fuses with a lysosome to form an autolysosome. Blockade of autophagy at this step would also result in an increased number of autophagosomes. In order to distinguish these possibilities, fusion of autophagosomes with lysosomes was examined by immunofluorescence. Co-localization of GFP-LC3 dots and signals for LAMP1, a lysosomal marker, was evaluated in the liver after CLP. As shown in Figure 2A, increased co-localization of LAMP1 and GFP-LC3 was observed in the CLP group compared with the sham-operated group at both 6 h (7.0 ± 3.1 in CLP versus 0.5 ± 0.7 in sham) and 24 h (10.7 ± 4.2 in CLP versus 2.2 ± 1.6 in sham; Figure 2B). At 6 h after CLP, 25.4% of GFP-LC3 dots were co-localized with LAMP1 signals, and this percentage increased to 58.8% by 24 h after CLP (Figure 2B). To evaluate autophagy flux (progression from autophagosome to autolysosome), the amount of p62 protein was examined [34,35]. As shown in Figure 2C, no significant difference was observed between the sham and CLP groups at either 6 or 24 h after the operation. However p62 protein significantly increased at 24 h compared to that at 6 h in CLP group.

**Figure 3:**
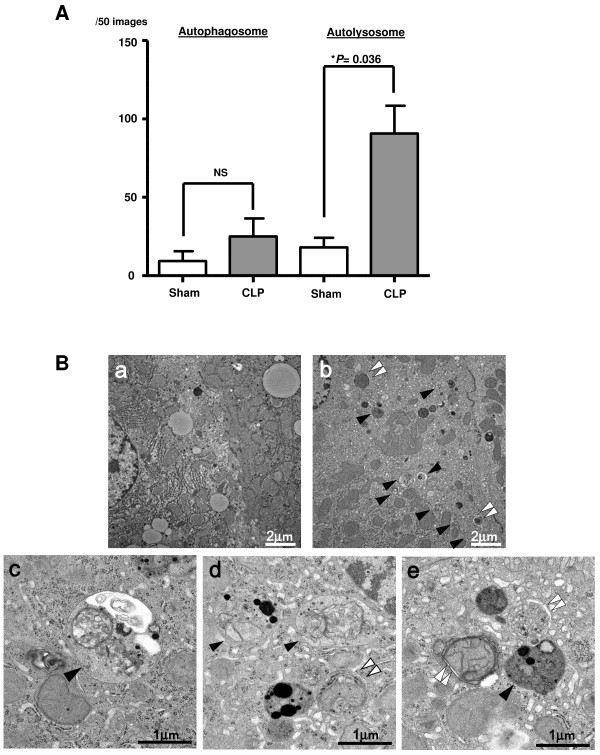
**Electron microscopic analysis of the liver. (A)** The number of autophagosomes and autolysosomes are compared in CLP and sham animals. All data are expressed as the mean ± SD. Data were analyzed for statistical significance using the Mann-Whitney test. Increase in autolysosomes in the CLP group was statistically significant (^*^P <0.05; n = 3); mean increases in autophagosomes in CLP compared to sham did not reach statistical significance. NS, not significant. **(B)** Images of electron microscopy of the liver; **a**: Liver sample obtained from sham-operated mice. Organelles in the hepatocyte are generally intact and lysosomes do not contain discrete membrane structures, although the inhomogeneous electron-dense material often seen in (hetero)lyosomes most certainly represent end-stage degradation of phospholipid and other cytoplasmic materials (a material at the light microscopic level referred to as lipofuscin); **b-e**: CLP-operated mice. Double arrow heads identify complex structures bounded by two membranes (autophagosomes); arrow heads identify single membrane-bound lysosomal complexes with degraded organellar content (autolysosomes); **e**: the double arrow head identifies an autophagosome that clearly contains an injured mitochondrion.

To further confirm the completion of autophagy, we examined liver samples by transmission electron microscopy. The autolysosome, which has a single limiting membrane and contains cytoplasmic/organellar materials at various stages of degradation, can be distinguished from the autophagosome (containing a double limiting membrane) by electron microscopy. The increase in autolysosomes in hepatocytes from sham versus CLP mice (25.0 ± 11.5 versus 90.7 ± 17.8) per 50 images for each mouse was statistically significant (*P* = 0.036) 6 h after CLP (Figure 3A,B). These data indicated that the autophagy process is completed in sepsis, rather than blocked at the fusion step, consistent with the immunofluorescence results. Importantly, despite an increased number of autophagosomes in septic samples, hepatocytes did not appear to be committed to cell death and the vast majority of mitochondria in both sham and CLP groups appeared normal (Figure 3B).

### Protective role of autophagy in the CLP septic model

**Figure 4:**
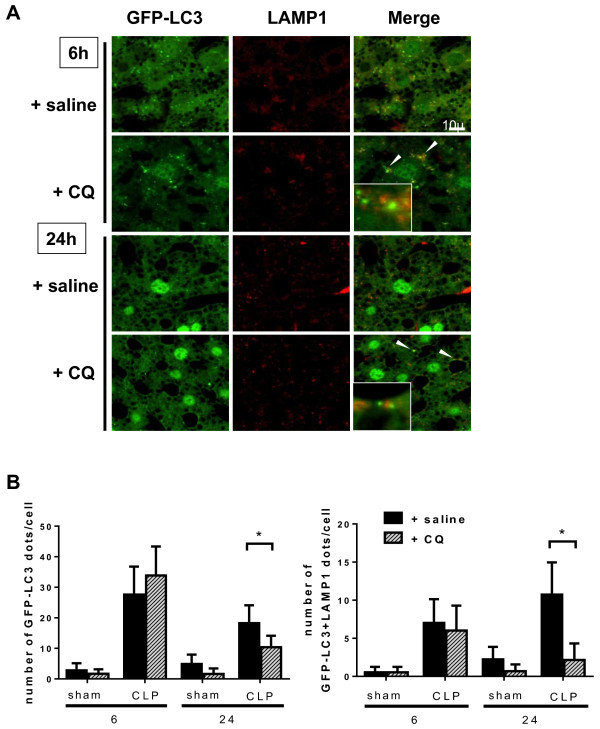
**Blockade of the autophagic process by chloroquine.** Green fluorescent protein- microtubule-associated protein light chain 3 (GFP-LC3) transgenic mice were administered saline or chloroquine (CQ) (60 mg/kg intraperitoneally) at 1 h after CLP or sham surgery. Samples were obtained from 6 or 24 h after surgery. **(A)** Confocal images of liver samples obtained from GFP-LC3 transgenic mice with or without CQ treatment. Blockade of autophagic process is indicated by the arrow head. **(B)** The number of GFP-LC3 or GFP-LC3 + lysosome-associated membrane protein type 1 (LAMP1) dots per cellular confocal image obtained from GFP-LC3 transgenic mice with or without CQ treatment was quantified at 6 and 24 h after cecal ligation and puncture (CLP). All data are expressed as the mean ± SD. Data were analyzed for statistical significance using the Student-Newman-Keuls test. Differences in the number of GFP-LC3 or GFP-LC3 + LAMP1 dots in CLP mice at 24 h compared with CLP mice at 24 h were statistically significant (^*^P <0.05; n = 50 cells /animal; n = 4 animals).

Since the autophagy machinery is activated after CLP, we examined whether this activation is beneficial or detrimental by inhibiting autophagy. Chloroquine, used primarily as an antimalarial drug, inhibits fusion of the autophagosome and lysosome by increasing autophagosomal and lysosomal pH [36,37]. We first confirmed that chloroquine (administered intraperitoneally 1 h after CLP, to a total dose of 60 mg/kg) suppressed autophagy in our CLP model. With chloroquine treatment, the number of GFP-LC3 dots and co-localized GFP-LC3 and LAMP1 were reduced after 24 h when compared to untreated animals in both CLP and sham-operated cohorts (Figure 4). Thus, chloroquine treatment suppressed the fusion of autophagosomes and lysosomes.

**Figure 5:**
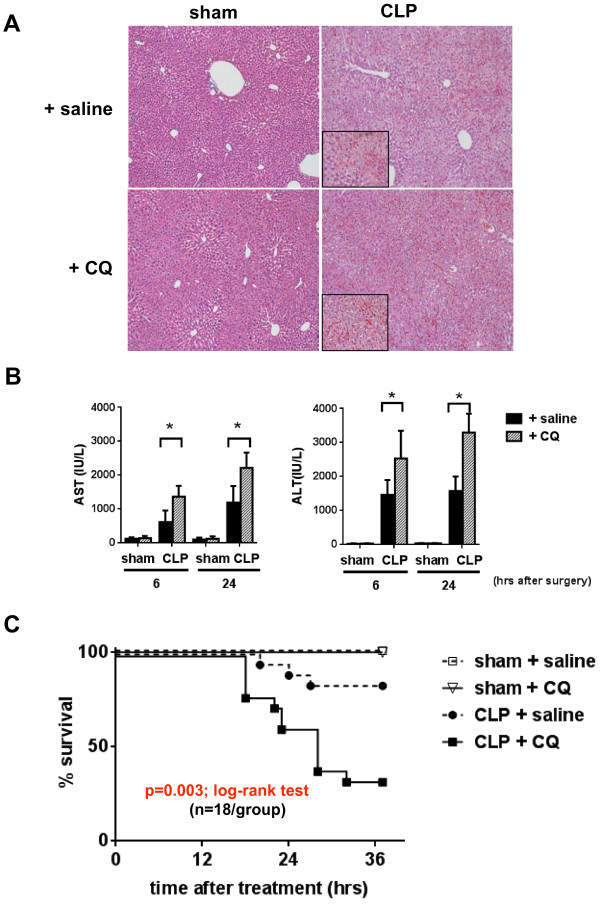
**Inhibition of autophagy enhances cecal ligation and puncture (CLP)-induced liver injury. (A)** Histological findings of mouse liver by hematoxylin and eosin staining (original magnification 100×) with a larger view (insets, 400×). Liver tissue was obtained from either sham-operated or CLP mice with or without chloroquine (CQ) treatment 6 h after surgery. **(B)** Hepatocellular damage as defined by serum aspartate aminotransferase (AST) and arginin aminotransferase (ALT) levels. Samples were obtained from either sham-operated or CLP mice with or without CQ treatment 6 and 24 h after surgery (n = 7/group). Data are shown as mean ± SD. ^*^P <0.05 versus saline treatment (one-way analysis of variance); individual group means were compared using the Student-Newman-Keuls test. **(C)** Survival after CQ or sterile saline administration (n = 18/group) in sham-operated or CLP mice; 3/18 mice in the CLP + saline group and 12/18 mice in the CLP + CQ group were sacrificed because they were moribund. Comparisons of overall survival were performed using the log-rank test (*P* = 0.003; CLP + saline versus CLP + CQ).

We next evaluated liver injury by histology and serum transaminase levels. In sham-operated mice with chloroquine treatment, no liver damage was observed. In contrast, we observed mid-zonal sinusoidal congestion and dilatation at 6 h after CLP. The congestion and dilatation became greater in CLP mice given chloroquine treatment, and was associated with subsequent liver dysfunction (Figure 5A). Serum AST and ALT were modestly increased at 6 and 24 h after CLP, but was significantly elevated compared to sham and untreated CLP animals after treatment with chloroquine (Figure 5B).

Finally, we examined the survival of CLP mice treated with or without chloroquine. Mice with labored breathing were considered moribund and were euthanized. Up to 36 h after CLP, the number of moribund mice in the chloroquine-treated group was significantly greater than that in the untreated group (Figure 5C; *P* = 0.003). From these data, it is evident that suppression of autophagy accelerates liver injury, and likely contributes to the increased mortality in the CLP septic model, thus suggesting that induction of autophagy plays a protective role against sepsis in this model.

## Discussion

In this study, we investigated the kinetics and role of autophagy in septic C57BL/6N mice over a 24-h period following CLP. We augmented our analysis by taking advantage of the unique characteristics of CLP-treated GFP-LC3 transgenic mice, in which LC3-positive autophagosomes can be directly visualized by GFP. Autophagosome formation as assessed by LC3-I/LC3-II conversion and GFP-LC3 dots was detected in liver, heart, and spleen, peaking at 6 h after CLP. These findings are corroborated by other recent reports of increased autophagy in the heart, liver, and lungs of both CLP-treated animals and in patients with sepsis [20-22,28]. Importantly, the time sequence of autophagy in these studies, with peak autophagosome formation at 6 to 8 h after CLP [20,22], is also compatible with our observations.

Autophagy is a complicated and dynamic multi-step process. Both an increase in autophagic flux and blockade of the downstream steps in autophagosomal maturation and lysosomal fusion may result in an increased number of autophagosomes. Thus, monitoring autophagic structures at different stages is necessary for accurate evaluation of this process. Indeed, it has been a point of some controversy in the literature whether the process of autophagy, culminating in fusion of the autophagosome with a lysosome, is completed or blocked after CLP. We believe we have resolved this matter. Our results, using two independent measures, clearly indicate that autophagy proceeds to completion in the liver after CLP. First, fusion of the autophagosome and lysosome was directly visualized using GFP-LC3 dots and LAMP1 immunofluorescence. Our data indicated that the absolute number of co-localized GFP-LC3 and LAMP1 signals continued to increase up to 24 h after CLP, and that LAMP1-co-localized GFP-LC3 signals as a percentage of total GFP-LC3 also increased to 64% by 24 h after CLP, indicating that the ongoing process of autophagy was proceeding to completion (unhindered formation of autolysosomes). To our knowledge, this is the first report to determine the dynamic changes in induction and completion of autophagy using co-localized GFP-LC3 and LAMP1 signals in the CLP model of sepsis.

Second, we analyzed samples by electron microscopy, perhaps the most reliable method for detecting autophagic structures. The number of autolysosomes in hepatocytes increased markedly after CLP compared to samples from sham-operated mice. These observations corroborate our earlier ultrastructural observations in CLP-treated mice and septic human patients [28]. Stated simply, autophagy is enhanced in hepatocytes by CLP-induced sepsis and proceeds to completion, at least in the earlier stages of sepsis.

A recent report by Chien and colleagues suggests that suppression or blockade of the autophagic process may occur at 18 h or later following CLP [22]. These observations conflict with our findings that autolysosome formation increases in the liver up to 24 h after CLP. To explore possible explanation for this discrepancy, we examined the amount of p62 protein, a marker for autophagy flux, in the liver. There were no statistically significant differences in the amount of p62 between sham and CLP groups at either 6 h or 24 h after the operation. Nonetheless, we observed a statistically significant increase in p62 protein at 24 h compared to 6 h in the CLP group, in spite of the increased autolysosome formation. Based on our observations, given the role of p62 in selective autophagy, we believe that rapid turnover of autophagy is required in sepsis to remove damaged organelles from injured cells and that the rate of autophagy may not be sufficient to deal with the extent of the damage in the liver. Because of the limited number of methods reported for monitoring autophagy flux *in vivo*, further study of a combination of other sophisticated assays is required. It has also been reported that fusion of autophagosomes with lysosomes is impaired in the heart and lung by 24 h after CLP [20,24]. We cannot directly respond to these data, but accept the possibility that the kinetics of autophagy are different for each organ. Indeed, Hsiao *et al*. demonstrated that autophagy is transiently activated in the kidney at 3 h after CLP, but declines from 6 h to 18 h as assessed by LC3-II expression [38]. It is also possible that different experimental conditions, such as the needle used for CLP, the amount and type of water and food intake after surgery, the intestinal microbiomes of the subject animal, and the housing conditions of the animals before and after surgery (for example, room temperature, humidity, and noise) may influence the results. Another possible explanation for this discrepancy may be the use of GFP-LC3 transgenic mice to monitor this process. The recent study by Lo *et al*. demonstrates that overexpression of LC3 protein facilitates the process of autophagy in the lung in a CLP model [24]. These data suggest that the amount of LC3 protein might be the rate-limiting factor. Further study to analyze baseline LC3 quantities in sham and GFP-LC3 mice may help resolve this matter.

It is generally accepted that autophagy promotes survival by supporting metabolism and mitigating damage by eliminating debris at the cellular level [6,19]. Blockade of autophagy by chloroquine resulted in liver dysfunction accompanied by an increase in serum AST and ALT at 6 and 24 h after CLP. Taken together, these findings support our survival data and suggest that the liver plays a key role during sepsis. Hepatocytes contribute to host defense by upregulating inflammatory responses by production of IL-6, C-reactive protein, fibrinogen, and thrombin. On the other hand, hemodynamic changes and excessive levels of inflammatory cytokines in early sepsis likely cause liver damage. Interestingly, induction of autophagy protects against the hepatotoxicity of acetaminophen [39] and ethanol [31]. In the latter setting, removal of damaged mitochondria by autophagy may be responsible for preventing hepatic cell apoptosis [31]. Previous reports also indicated that hepatocyte resistance to injury by oxidative stress is mediated by autophagy [27], and that impaired autophagy may promote oxidative-induced liver injury associated with over-activation of the JNK signaling pathway that induces cell death [40]. In the liver, autophagy is important for maintaining the balance of energy and nutrients for cell functions, removal of misfolded proteins, resistance to oxidative stress [40], and turnover of mitochondria under both normal and physiological conditions. Thus, disturbance of autophagy in the liver could have a major impact on liver physiology and disease [41-43]. Our data suggest that suppression of autophagy by chloroquine after CLP is in fact detrimental. Histological examination of the liver revealed that mid-zonal sinusoidal congestion and dilatation became greater in CLP operated mice given chloroquine treatment compared to untreated mice. However, no evidence of hepatocellular necrosis was observed in the chloroquine treatment group at 6 or 24 h after the operation. We believe that the primary effect of autophagy inhibition in hepatocytes is to prevent damaged organelles such as mitochondria from being targeted for autophagic clearance. Although chloroquine has pleiotropic pharmacological activities and is not a specific inhibitor of autophagy, it nonetheless selectively interferes with autophagosome/lysosome fusion (presumably by increasing lysosomal pH). Even so, it remains unclear from our observations how autophagy in hepatocytes plays a protective role against CLP-induced liver dysfunction and overall survival, since suppression of autophagy by chloroquine is not liver-specific. Perhaps the role of autophagy in CLP-induced sepsis in each organ will be clarified by using organ-specific autophagy-conditional knockout mice.

Several reports have demonstrated that induction of autophagy by other pharmacological agents, such as rapamycin, improves cardiac function and inflammatory responses in CLP mice [20,24,44-46]. However, since there are no autophagy-specific inhibitors or inducers available at this time, we must be careful in interpreting these data. Nevertheless, activation of autophagy could be a potential therapeutic target in sepsis, since our data suggest that induction of autophagy in the early phase of sepsis may support immunomodulation. Recent data measured by ICU resource use and infection rates indicate that early parenteral nutrition in critically ill patients is harmful [47]. We might infer, then, that induction of autophagy by means of nutrient deprivation in the acute phase of sepsis may be beneficial, particularly for those patients with signs of severe sepsis.

## Conclusions

In conclusion, we have shown that autophagy is induced in several organs in the first 24 h after CLP in an animal model of sepsis, and that the entire process of autophagy, from early envelopment of damaged cytosolic elements to fusion of autophagosomes with lysosomes, is activated in liver. We also conclude that autophagy plays a protective role in organ dysfunction during sepsis. Development of specific modulators of autophagy and the means to monitor autophagy in real time will be critical to the successful introduction of pro-autophagic therapies to the field of critical care medicine.

## Key messages

• All intact autophagy-related processes are activated rather than suppressed in liver in a mouse CLP-induced sepis model.

• Autophagy plays a protective role against sepsis.

## Abbreviations

ALT: Arginin aminotransferase; AST: Aspartate aminotransferase; CLP: Cecal ligation and puncture; GFP: Green fluorescent protein; IgG: Immunoglobulin G; IL: Interleukin; LAMP1: Lysosome-associated membrane protein type 1; LC3: Microtubule-associated protein light chain 3; OCT: Optimum cutting temperature; PBS: Phosphate-buffered saline; PF A: Paraformaldehyde; TBS: Tris-buffered saline.

## Competing interests

The authors declare that they have no competing interests.

## Authors’ contributions

WT carried out the experiments, analyzed results, prepared the figures, and wrote the manuscript. EW conceived the study concept, and participated in its design and grant finding, performed electron microscopy analysis, and wrote the manuscript. LF and HW-T performed the animal experiments and protein analysis. HY performed histological analysis of the liver and critical review of the manuscript. PES participated in analysis of electron micrographs and critical review of the manuscript. TT participated in the design of the study, and gave a critical suggestion to the project. SO directed the project and secured funding. MH conceived the study concept, participated in its design, and wrote the manuscript. All authors read and approved the final manuscript.
